# The Impact of Metastatic Brain Lesions on Enhanced Recovery After Surgery Protocols for Elective and Emergent Supratentorial Brain Tumor Surgery: A Retrospective Review

**DOI:** 10.1227/neuprac.0000000000000177

**Published:** 2025-10-13

**Authors:** Harshal Shah, John L. Kilgallon, Geoffrey O'Malley, Syed Sarwar, Travis R. Quinoa, Nitesh V. Patel

**Affiliations:** * Department of Neurosurgery, Hackensack Meridian School of Medicine, Nutley, New Jersey, USA;; ‡ Department of Neurosurgery, HMH-Jersey Shore University Medical Center, Neptune, New Jersey, USA

**Keywords:** Craniotomy, ERAS, Metastasis, Tumor

## Abstract

**BACKGROUND AND OBJECTIVES:**

Enhanced Recovery After Surgery protocols are increasingly being used in neurosurgery, with multiple postoperative day (POD) zero and POD1 discharge protocols being implemented for patients undergoing craniotomies for brain tumor resection. We discuss our experience with implementing a POD1 protocol for patients both admitted electively and from the emergency department (ED).

**METHODS:**

We conducted a retrospective cohort study of 61 consecutive patients treated with craniotomies for brain tumor resection at Jersey Shore University Medical Center by a single surgeon from August 2022 to April 2024.

**RESULTS:**

A total of 39 patients met the inclusion criteria for POD1 discharge protocol. Of these 33.3% (n = 13) were successfully discharged on POD1, whereas 38.5% (n = 15) were cleared by neurosurgery on POD1 but required additional management before discharge. There were 7 ED visits and readmissions within 30 days, of which 3 were neurosurgery related. One patient had a planned reoperation, another patient had rapid tumor recurrence, and the third patient had symptomatic hyponatremia. There were no statistically significant differences in hospital stay between patients admitted electively vs through the ED. The only statistically significant factor was metastatic disease. Specifically, patients with metastatic disease had significantly longer hospital stays (t = 2.14 days, *P* = .04) and longer discharge delays (t = 2.25 days, *P* = .04) compared with those with nonmetastatic disease.

**CONCLUSION:**

We found no statistically significant difference between elective and ED admitted patients regarding hospital length of stay or discharge delay, suggesting that both cohorts can successfully participate in this protocol. The only significant delaying factor was metastatic disease, with many patients presenting with brain tumors as the first sign of underlying malignancy. We suggest that for patients with metastatic disease to the brain, only patients with established cancer diagnoses be included in these Enhanced Recovery After Surgery protocols.

**ABBREVIATIONS:**
ED: emergency department
ERAS: enhanced recovery after surgery
KPS: Karnofsky Performance Status
LOS: length of stay
pLOS: postoperative LOS.

Enhanced recovery after surgery (ERAS) is a model of care introduced in 1997 focused on reducing postoperative complications and improving rehabilitation.^1^ ERAS has been implemented in many surgical subspecialities and has shown to significantly reduce hospital length of stay (LOS) without increases in postoperative complications, while decreasing healthcare costs for patients.^2^ One of the growing applications of ERAS protocols in neurosurgery has been for patients undergoing craniotomies, with studies showing that application of these protocols for craniotomies can safely and effectively reduce patient LOS and healthcare costs with no significant differences in readmission rates.^3,4^

Within craniotomies, one of the major applications of ERAS protocols has been craniotomies for brain tumor resections. These patients are especially important to target because they have a greater risk of decreased postoperative cognitive function and functional status, emphasizing the importance for their safe but swift hospital discharge.^5^ Multiple studies have successfully implemented same day discharge protocols for patients undergoing elective craniotomies with no significant increases in readmission or postoperative complication rates.^6-12^ A major takeaway from many of these studies is the careful selection of patients that should qualify for the protocol.

The purpose of this study was to retrospectively assess outcomes for patients with cranial tumors who underwent treatment and surgical resection from August 2022 through February 2024 using an ERAS postoperative day (POD) 1 discharge protocol. Our protocol was modeled after the protocol used by Levy at al.^6^ in their same day discharge study. Preoperatively, patients are counseled on pain expectations, discharge timing, and steps of care. Intraoperatively, there is a focus on linear incisions, minimal hair shaving, and avoiding unnecessary wound dressings. Postoperatively, there is a focus on early diet introduction and getting out of bed, restricting excessive pain medications, early postoperative imaging, and repeat counseling on the goal for POD1 discharge. Unlike many of the other studies implementing these protocols, we assessed both elective patients and patients admitted from the emergency department (ED) for protocol eligibility and participation. Thus, the goal of this study was to assess the efficacy of the ERAS protocol in reducing hospital LOS in both elective and ED patients as well as to identify any factors such as diagnosis and tumor location that may be associated with delayed hospital discharge.

## METHODS

This retrospective cohort study was performed for 61 consecutive patients treated with craniotomies for brain tumor resection at Jersey Shore University Medical Center by a single surgeon from August 2022 to April 2024. The study was approved by the Institutional Review Board before any patient records were extracted. Patient consent was obtained before all procedures. Of the 61 patients, 39 patients met the inclusion criteria for the protocol. Inclusion criteria were supratentorial lesions, an American Society of Anesthesiologists (ASA) class ≤4, and a preoperative Karnofsky Performance Status (KPS) score ≥70. Exclusion criteria were an EBL >300 mL and an estimated operative time >3 hours. Data were extracted by 3 authors. ERAS protocol implementation began with preoperative counseling by the neurosurgeon and neurosurgical staff in office or in the hospital for patients admitted from the ED. Intraoperative protocol components were implemented by the neurosurgeon, and postoperative components were performed by the multidisciplinary care team including but not limited to the neurosurgeon, neurosurgical hospital team of physician assistants, nursing staff, and physical and occupational therapy. Information on patients' demographic characteristics, risk factors and comorbid conditions, admit status (ED admission vs elective surgery), insurance status, ASA and KPS scores, tumor size and location, previous treatments, and presenting complaints were extracted. Procedural variables extracted were length of surgery, estimated blood loss, awake status, and extent of resection. Postoperative variables extracted were postoperative complications, hospital LOS, day of neurosurgery clearance, ED and hospital readmissions, and KPS scores at first follow-up. Discharge delay was calculated as the difference between day of neurosurgery clearance and day of discharge.

Simple linear regression analyses were conducted to assess the impact of Diagnosis, Predominant Tumor Location, and Side of Lesion on LOS variables, including Hospital Admission Length After Surgery, Discharge Delay, and intensive care unit Admission Length After Surgery. An independent *t*-test was also performed to compare patients admitted electively vs those admitted from the ED regarding discharge delay and postoperative LOS (pLOS). A *P*-value of <.05 was considered statistically significant. Statistical analysis was performed using Python (version 3.8.0, Python Software Foundation). An analysis of variance was conducted to determine whether there were statistically significant differences in discharge delay and pLOS across different diagnoses.

## RESULTS

**FIGURE 1.**
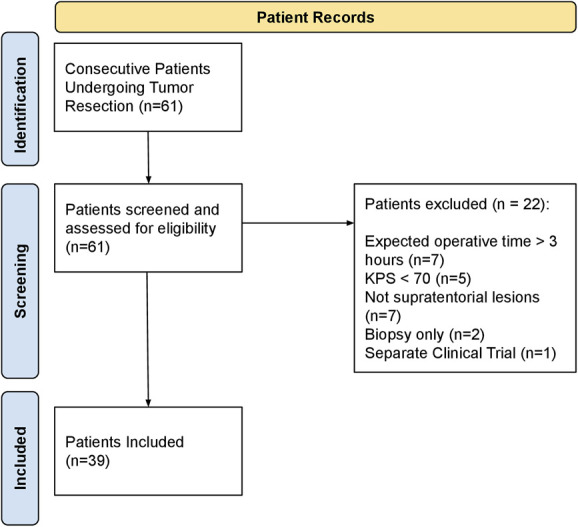
Patient inclusion and exclusion summary. KPS, Karnofsky Performance Status.

**FIGURE 2.**
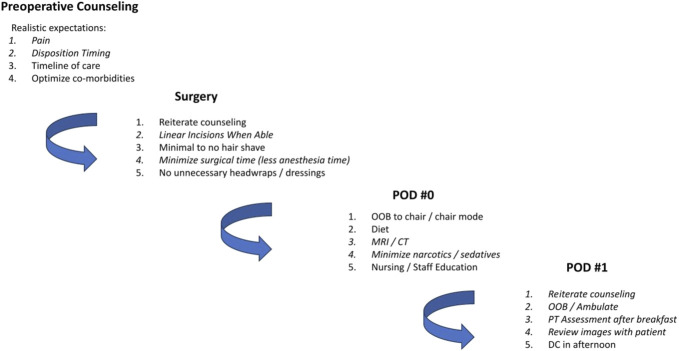
POD1 discharge protocol summary. CT, computed tomography; DC, discharge; OOB, out of bed; POD, postoperative day.

A total of 61 patients underwent craniotomies for brain tumor resection, of whom 39 met the inclusion criteria. The reasons for exclusion included expected operative time >3 hours (n = 7), infratentorial lesions (n = 7), KPS <70 (n = 5), biopsy only (n = 2), and separate clinical trial (n = 1) (Figure 1). A summary of the POD1 protocol is shown in Figure 2. A summary of patient and tumor characteristics is shown in Table 1. Among the participants, 56.4% were female (n = 22). A total of 23.1% (n = 9) had recurrent tumors; 28.2% (n = 11) had previous radiotherapy, 3.1% (n = 9) had previous chemotherapy, and 10.3% (n = 4) had undergone previous operations. Regarding discharge disposition, the majority (84.6%, n = 33) were discharged home, with 7.7% (n = 3) discharged to a rehabilitation facility, 5.1% (n = 2) to a nursing facility, and 2.5% (n = 1) to hospice care.

**TABLE 1.** Patient and Tumor Characteristics

Variable	Value
Sample size	39 (100%)
Male	17 (43.6%)
Female	22 (56.4%)
History of psychiatric diagnoses	11 (28.2%)
Recurrent tumor	9 (23.1%)
Previous operation	4 (10.3%)
Previous chemotherapy	9 (23.1%)
Previous radiotherapy	11 (28.2%)
Discharge disposition	
Home	33 (84.6%)
Rehabilitation facility	3 (7.7%)
Nursing facility	2 (5.1%)
Hospice	1 (2.5%)
Tumor location	
Left	21 (53.8%)
Right	18 (46.2%)
Frontal	15 (38.5%)
Parietal	15 (38.5%)
Temporal	7 (17.9%)
Occipital	1 (2.6%)
Other/multifocal	1 (2.6%)
Mean tumor volume (cm^3^)	64.9 (SD 68.6)
Diagnosis	
Metastatic disease	16 (41.0%)
Meningioma	9 (23.1%)
Glioblastoma	7 (17.9%)
High-grade glioma	3 (7.7%)
Low-grade glioma	1 (2.6%)
Oligodendroglioma	1 (2.6%)
CNS lymphoma	1 (2.6%)
DNET	1 (2.6%)
Median KPS	80
Median American Society of Anesthesiologists class	III

CNS, central nervous system; DNET, dysembryoplastic neuroepithelial tumor; KPS, Karnofsky Performance Status.

Tumor location was fairly evenly distributed between the left (53.8%, n = 21) and right (46.2%, n = 18) sides. Anatomically, 38.5% (n = 15) of tumors were located in the frontal region, another 38.5% (n = 15) in the parietal region, 17.9% (n = 7) in the temporal region, and 2.6% (n = 1) each in the occipital region and other/multifocal areas. Regarding diagnoses, metastatic disease was the most common (41.0%, n = 16), followed by meningioma (23.1%, n = 9), glioblastoma (17.9%, n = 7), high-grade glioma (7.7%, n = 3), and less common diagnoses such as low-grade glioma, oligodendroglioma, central nervous system lymphoma, and dysembryoplastic neuroepithelial tumor (each 2.6%, n = 1). The median KPS was 80, and the median ASA class was III.

The pLOS, time between neurosurgery clearance and discharge, 30 day ED visits, 30 day hospital readmissions, and median KPS at first follow-up are shown in Table 2.

**TABLE 2.** Postoperative Outcomes and 30 d ED Visits and Readmissions

Variable	Outcome
Mean pLOS	3.2 d
Mean Discharge delay	1.6 d
Discharged POD1	13 patients (33.3%)
Cleared POD1	15 patients (38.5%)
Postoperative complications	1 (2.6%)
30 d ED Visits	7 (17.9%)
30 d Hospital Readmissions	8 (20.5%)
30 d Neurosurgery Readmissions	3 (7.7%)
Median KPS at 1st Follow-Up	90

ED, emergency department; KPS, Karnofsky Performance Status; pLOS, postoperative length of stay; POD, postoperative day.

**FIGURE 3.**
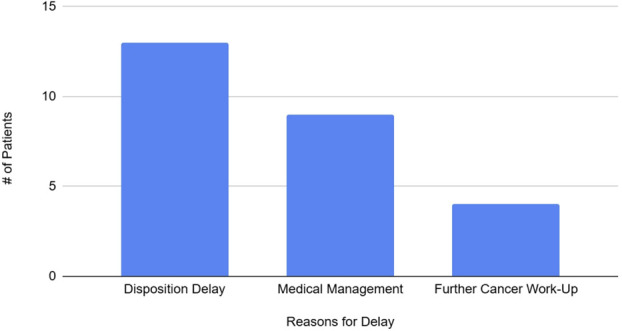
Reasons for discharge delay.

Overall, 1 patient had a postoperative complication of a seizure. There were no other postoperative complications. The average hospital LOS was 3.2 days, and the average delay between neurosurgery clearance and hospital discharge was 1.6 days. The reasons for discharge delay are shown in Figure 3, with the most prominent being disposition delay. Disposition delay included patients who required further time for safe discharge planning (for example, waiting for rehabilitation bed) or discharge delays for further medical work up and management. Thirteen patients (33%) were successfully discharged on POD1, and 15 patients (38%) were cleared by neurosurgery on POD1. Seven patients had ED visits within 30 days, and all these patients were readmitted to the hospital. Reasons for ED visits included sepsis, pulmonary embolism, aphasia, pneumonia, lower extremity weakness, right eye pain, and surgical site bleed. Three patients had neurosurgical readmissions, with 1 patient requiring an additional operation for tumor recurrence.

Further stratification was performed based on admission status. There were 14 patients (35.9%) admitted electively for same day surgery, with an average pLOS of 3.6 days and average delay of 1.4 days. There were 3 patients (21%) discharged on POD1 and 3 (21%) were cleared on POD1. A total of 25 patients (61.4%) were admitted from the ED, with an average pLOS of 2.9 days and average delay of 1.7 days; 10 patients (40%) were discharged on POD1, and 13 patients (48%) of patients were cleared on POD1. Of the 9 hospital readmissions within 30 days, 5 patients were from the elective group and 4 patients were from the ED group. A *t*-test was performed to compare the elective vs ED admit groups, and it showed no significant difference regarding hospital LOS (t = 0.80, *P* = .43; 95% CI [-1.07, 2.43]) or discharge delay (t = -0.44, *P* = .66; 95% CI [-1.60, 1.09]).

The patients were also divided based on diagnosis and tumor location, with their LOS and delays shown in Table 3. An analysis of variance was performed to determine whether tumor characteristics or diagnosis were predictive factors in longer LOS and discharge delays. The first analysis compared the side of the lesion (left vs right) and found no significant differences for discharge delay (F = 0.19, *P* = .83) or LOS (F = 1.35, *P* = .27). An additional analysis was performed looking at which brain lobe was involved which also found no statistically significant differences for discharge delay (F = 0.49, *P* = .74) and LOS (F = 0.39, *P* = .82). The final analysis compared different diagnoses and also found no significant differences on discharge delay (F = 0.83, *P* = .57) and LOS (F = 0.84, *P* = .56). Because of the wide distribution of diagnoses, an independent *t*-test was performed comparing patients with metastatic disease and primary central nervous system tumors. The results revealed a statistically significant difference in both hospital admission length and discharge delay between the 2 groups. Specifically, patients with metastatic disease had significantly longer hospital stays (t = 2.14, *P* = .04, 95% CI [0.069, 3.69]) and longer discharge delays (t = 2.25, *P* = .04, 95% CI [0.13, 3.38]) compared with those with nonmetastatic disease. In addition, patients with nonmetastatic disease were 77% less likely to have a discharge delay compared with those with Metastatic Disease (odds ratio = 0.23).

**TABLE 3.** Average Hospital Length of Stay and Discharge Delay by Diagnosis and Tumor Characteristics

Variable	Hospital length of stay after surgery, d (range)	Discharge delay, d (range)
Diagnosis		
CNS lymphoma	5 (N/A)	1 (N/A)
DNET	2 (N/A)	0 (N/A)
Glioblastoma	2.3 (1-4)	0.9 (0-5)
High- grade glioma	1.7 (1-2)	0.3 (0-1)
Low-grade glioma	2 (N/A)	1 (N/A)
Meningioma	2.7 (1-8)	1.1 (0-4)
Metastatic disease	4.3 (1-10)	2.6 (0-9)
Oligodendroglioma	2 (N/A)	1 (N/A)
Tumor location		
Frontal	3.3 (1-10)	1.3 (0-8)
Occipital	2 (N/A)	1 (N/A)
Other/multifocal	1 (N/A)	0 (N/A)
Parietal	3.6 (1-10)	2.2 (0-9)
Temporal	2.5 (1-8)	1.1 (0-5)
Side of lesion		
Left	3.1 (1-10)	1.6 (0-9)
Right	3.2 (1-10)	1.5 (0-8)

CNS, central nervous system; DNET, dysembryoplastic neuroepithelial tumor.

## DISCUSSION

The results of our analysis show that there were no statistically significant differences between ED and elective patients regarding LOS or discharge delay. In addition, the only predictive factor for patients having a longer LOS and delay was metastatic disease. For many of these patients, the metastatic brain tumor was the first presentation of their underlying cancers, and as a result they often required additional workup in the hospital to identify and begin treatment planning for their cancer before discharge. We recommend that metastatic patients be carefully considered before enrollment into these protocols. Within our patient cohort, 16 patients had metastatic disease, and only 4 patients were successfully discharged on POD1. All 4 of these patients had established diagnoses and had been treated previously by chemotherapy and/or radiotherapy for their underlying cancer. And so, we suggest that for patients with metastatic disease, only patients with a pre-existing diagnosis and ongoing/completed cancer treatment be considered for enrollment in a POD1 discharge protocol.

Discharge delays made up for a significant portion of our patients, with 15 patients cleared on POD1 by neurosurgery but not being discharged until later. Two of those patients had metastatic disease and required further cancer workup after surgery. One patient required additional imaging for cranioplasty planning, which was also 1 of the 2 patients that required reoperation after initial resection. Rehabilitation facility placement delay occurred in 2 of the patients. One patient required orthopedic surgery for a femur fracture that extended hospital stay. The patient was a trauma patient with a brain tumor found incidentally on computed tomography head imaging. Two patients were discharged on POD2 because of delays in physical therapy and occupational therapy evaluations. Four patients had delays from other consult services. Two patients had symptoms requiring additional management, with 1 patient having postoperative urinary retention and another patient with underlying cardiac disease experiencing symptomatic bradycardia requiring additional cardiology management. The final patient felt more comfortable staying in the hospital an additional delay and was discharged on POD2.

Rehabilitation facility placement led to discharge delays in all 3 patients. Addressing these delays is very important for patient recovery, because it delays their ability to access resources for recovery. In addition, delays discharging to rehabilitation facilities have been associated with increased LOS at rehabilitation facilities and decreased rehabilitation efficiency.^13^

A total of 11 patients were not cleared on POD1 by the neurosurgery team. Six patients were cleared on POD2. Four patients experienced symptoms that required an additional hospital day for safe discharge. One patient required an additional day of electroencephalogram monitoring to rule out a potential seizure which was negative. The final patient had pre-existing dysphagia requiring total parenteral nutrition. She was admitted to the hospital for this dysphagia, with her tumor found incidentally on computed tomography imaging. One patient was cleared on POD3 because of hospital acquired pneumonia management. The final 4 patients were cleared on POD4. Two patients had severe comorbidities (hypertension, chronic kidney disease) that required extensive medical management. One patient had a postoperative seizure, and the final patient had pre-existing psychiatric conditions that were exacerbated postoperatively and required additional medication optimization by psychiatry for safe discharge. No patients were cleared later than POD4.

An important aspect to consider in our evaluation of the safety of this POD1 discharge protocol is evaluating the 30 day ED visits and hospital readmissions. There were 7 patients that had 30-day ED visits, and all 7 patients were readmitted to the hospital. There was 1 additional 30 day hospital readmission which was for a planned chemotherapy treatment. Of the 7 patients, 3 had neurosurgical related readmissions. One patient had a planned cranioplasty but presented through the ED, and another patient had rapid tumor recurrence requiring a second operation. The third patient had aphasia that was concerning seizures, but the patient was found to have symptomatic hyponatremia and electroencephalogram monitoring found no underlying seizure activity. The other 4 patients with hospital readmissions were because of pneumonia, sepsis, pulmonary embolism, and lower extremity edema. These patients did not require any neurosurgical interventions.

Our study also compared patients admitted from the ED with elective patients. No previous study had evaluated the use of the POD1 protocol for patients admitted from the ED. The only aspect of protocol implementation that differed for this group was some restriction with pre-operative comorbidity minimization such as smoking cessation. Otherwise, both patient groups benefitted from the same levels of pre-operative education and counseling on expected pain and care timelines. Although we have a small sample size of patients, our data showed no significant difference between the 2 groups. Of the 7 patients that had 30 day ED visits and readmissions, 4 patients were from the elective group and 3 patients were from the ED admit group. Although we need more patient data to further compare the 2 groups, our initial experience shows that these patients can also partake in the protocol.

### Limitations

This is a retrospective review of 39 patients who all underwent resection by the same surgeon and at the same institution. This is a small sample size, and there is potential for bias from a single surgeon and single institution experience.

## CONCLUSION

A total of 39 patients underwent craniotomies for brain tumor resection, of whom 33.3% (n = 13) patients were successfully discharged POD1, whereas 15 patients (n = 38.5%) were cleared by neurosurgery on POD1 but experienced discharge delays. We found no statistically significant difference between ED and elective patients regarding hospital LOS or discharge delay, suggesting that both cohorts can successfully participate in this protocol. The only factor that was significant for causing longer LOS and discharge delay were patients with metastatic disease. Although these patients can participate in this protocol, we suggest that only patients with established cancer diagnoses who are already being treated be included to mitigate further delays.
